# Intraindividual Embryo Morphokinetics Are Not Affected by a Switch of the Ovarian Stimulation Protocol Between GnRH Agonist vs. Antagonist Regimens in Consecutive Cycles

**DOI:** 10.3389/fendo.2020.00246

**Published:** 2020-04-28

**Authors:** Jens E. Dietrich, Alexander Freis, Franziska Beedgen, Kyra von Horn, Verena Holschbach, Julia Liebscher, Thomas Strowitzki, Ariane Germeyer

**Affiliations:** ^1^Department of Gynecologic Endocrinology and Fertility Disorders, Heidelberg University Women's Hospital, Heidelberg, Germany; ^2^Faculty of Veterinary Medicine, Justus-Liebig-University Giessen, Giessen, Germany

**Keywords:** human embryology, time-lapse imaging, morphokinetics, GnRH agonist, GnRH antagonist

## Abstract

**Background:** The impact of controlled ovarian stimulation (COS) during medically assisted reproduction (MAR) on human embryogenesis is still unclear. Therefore, we investigated if early embryonic development is affected by the type of gonadotropin-releasing hormone (GnRH) analog used to prevent a premature LH surge. We compared embryo morphology and morphokinetics between GnRH agonist and antagonist cycles, both involving human chorionic gonadotropin (hCG)-trigger. To reduce possible confounding factors, we used intraindividual comparison of embryo morphokinetics in consecutive treatment cycles of the same patients that underwent a switch in the COS protocol.

**Methods:** This retrospective cohort study analyzed morphokinetics of embryos from patients (*n* = 49) undergoing a switch in COS protocols between GnRH agonists followed by GnRH antagonists, or vice versa, after culture in a time-lapse incubator (EmbryoScope®, Vitrolife) in our clinic between 06/2011 and 11/2016 (*n* = 49 GnRH agonist cycles with *n* = 172 embryos; *n* = 49 GnRH antagonist cycles with *n* = 163 embryos). Among time-lapse cycles we included all embryos of the two consecutive cycles before and after a switch in the type of COS in the same patient. *In-vitro* fertilization (IVF) or intracytoplasmic sperm injection (ICSI) was performed and embryos were imaged up to day 5. Data were analyzed using Mann-Whitney *U* test or Fisher's exact test. The significance level was set to *p* = 0.05. Patients with preimplantation genetic screening cycles were excluded.

**Results:** The mean age (years ± standard deviation) of patients at the time of treatment was 35.7 ± 4.3 (GnRH agonist) and 35.8 ± 4.0 (GnRH antagonist) (*p* = 0.94). There was no statistically significant difference in the number of oocytes collected or the fertilization rate. The numbers of top quality embryos (TQE), good-quality embryos (GQE), or poor-quality embryos (PQE) were also not different in GnRH agonist vs. antagonist cycles. We found no statistically significant difference between the analyzed morphokinetic parameters between the study groups.

**Conclusions:** Our finding supports the flexible use of GnRH analogs to optimize patient treatment for COS without affecting embryo morphokinetics.

## Introduction

The goal of preimplantation development is to form a blastocyst that is able to hatch and implant into the receptive endometrium (1). The implantation potential of embryos can be predicted by the evaluation of their morphology (2–4). Time-lapse imaging was introduced to enable the assessment of morphology and developmental kinetics (morphokinetics) in a continuous *in vitro* culture by acquiring images at a high temporal resolution in multiple focal planes (5, 6). Despite the increased number of parameters available for non-invasive assessment, the clinical benefit of time-lapse imaging is still controversial (7–10). The quality of scoring embryos is affected by inter- and intraindividual variability, making standardization of annotation, and nomenclature necessary (11, 12).

Embryo morphokinetics reflect the developmental competence of germ cells, and thus may be affected by patients' confounders, e.g., causes of infertility and comorbidities [e.g., polycystic ovary syndrome (PCOS) or endometriosis], female age and female smoking status (12–18). *In vitro* culture conditions, i.e., the type of culture media and gas composition used, might have an additional impact on embryo development (7, 19, 20).

Another possible confounder described in the literature is the protocol used for controlled ovarian stimulation (COS) (12, 21). Current COS protocols involve the application of GnRH analogs (agonists or antagonists) to inhibit the endogenous luteinizing hormone (LH) surge, gonadotropins to achieve multi-follicular growth, as well as an ovulation trigger (22). Thus, it is currently possible to choose from a variety of COS protocols for patient specific medically assisted reproduction (MAR) treatments (22, 23). The choice of the initial COS protocol is based on patient's medical characteristics, but also on the patient's and physician's preference (21, 23, 24). GnRH agonist protocols enable a flexible start of COS (23). GnRH antagonist protocols result in a lower rate of ovarian hyperstimulation syndrome compared to GnRH agonist protocols and are therefore favored in some conditions, e.g., if the woman has a high ovarian reserve (23, 24).

COS protocols act on follicle maturation and as a consequence may affect the developmental competence of oocytes, the resulting embryos and thus the clinical outcome of treatments (21, 25). In addition, GnRH may have an extrapituitary function, acting directly on embryos (26). Thus, GnRH analogs may also affect embryogenesis directly (27, 28). GnRH agonist protocols may lead to a more homogenous follicle recruitment compared to GnRH antagonist cycles (29). In GnRH antagonist protocols a higher rate of oocytes with cytoplasmic abnormalities were described (30). Muñoz et al. found that embryos cleaved faster when they were generated in cycles with GnRH antagonist plus GnRH agonist-trigger vs. GnRH agonist plus human chorionic gonadotropin (hCG)-trigger, but embryo quality was not affected (31). However, despite the described effects on oogenesis and embryogenesis, a recent intervention review found no difference between GnRH antagonist and the long protocol of GnRH agonists for COS regarding live birth and miscarriage rates (32).

As the influence of COS protocols on embryogenesis is still unclear, the aim of this study was to investigate if embryo quality and morphokinetics are affected by switching the type of GnRH analog used within the same patient. A switch in the COS protocol may be a favorable treatment approach for some patients, i.e., in cases of ovarian hyperstimulation syndrome, poor response in a treatment cycle or individual temporal factors.

In this study we compared embryo morphology and morphokinetics between GnRH agonist and antagonist cycles, both involving hCG-trigger. To reduce possible confounding factors, we compared intraindividual embryo morphokinetics in consecutive treatment cycles of the same patients that underwent a switch in the COS protocol.

## Materials and Methods

### Data Collection and Study Population

In this study, data of *n* = 49 patients attending the Department of Gynecological Endocrinology and Fertility Disorders, Ruprecht-Karls University Heidelberg, between June 2011 and November 2016 were retrospectively analyzed. Inclusion criteria were at least 2 treatment cycles with a switch in COS protocols between GnRH agonists followed by GnRH antagonists, or vice versa, with embryo culture in a time-lapse incubator (TLI, EmbryoScope®, Vitrolife). In total we analyzed *n* = 49 GnRH agonist cycles with *n* = 172 embryos and *n* = 49 GnRH agonist cycles with *n* = 163 embryos. Cycles with preimplantation genetic testing (PGT) were excluded. All embryos of the two consecutive cycles cultured in a TLI before and after a switch of COS protocols were analyzed. Here, “consecutive cycles” include only those with time-lapse imaging. Intervening cycles without time-lapse imaging were not considered.

### Controlled Ovarian Stimulation, Ovum Pick-Up

The choice of the initial stimulation protocol was based on individual patients' characteristics (e.g., PCOS, ovarian reserve, endometriosis), time available and preference of the patient and physician, e.g., women with PCOS received mostly the GnRH antagonist protocol, while women with endometriosis were more likely to receive the GnRH agonist protocol.

For COS using a long GnRH agonist protocol, patients received nafarelin (Synarela®, Pfizer, Berlin, Germany) at a dose of 0.4 mg daily or triptorelin (Decapeptyl®, Ferring, Kiel, Germany) 0.1 mg daily starting or leuprorelin (Enantone®, Takeda, Berlin, Germany) 3.75 mg once in the midluteal phase of the previous cycle (20th day of the cycle) as GnRH agonists. Pituitary suppression was confirmed by LH values < 5 U/l and estradiol [E2] values < 50 pg/ml after menstruation. Ovarian stimulation was initiated with follitropin α (Gonal-f®, Merck Serono, Darmstadt, Germany), follitropin β (Puregon®, MSD, Munich, Germany) at a dose of 100–300 IU/day or menotropin (Menogon®, Ferring, Kiel, Germany) at a dose of 150–300 IU/day, depending on multiple factors, such as age, weight, body mass index (BMI), antral follicle count (AFC), and Anti-Muellerian hormone (AMH). The dosage of the respective gonadotropin was adjusted, if necessary, after 5–6 days based on E2-levels and sonographic follicular development.

The GnRH antagonist protocol was performed as follows: after confirming normal ultrasound (endometrium thickness < 6 mm; no follicles > 10 mm) and basal hormone levels (E2 < 70 pg/ml), patients received the same gonadotropin stimulation as mentioned above starting on cycle day two or three. GnRH antagonists ganirelix (Orgalutran®, MSD, Munich, Germany) or cetrorelix (Cetrotide®, Merck Serono, Darmstadt, Germany) at a dose of 0.25 mg per day were additionally applied when the leading follicle reached a diameter of 14 mm and continued until the day of the induction of ovulation.

A consistent rise in E2 levels and follicle growth was monitored until the presence of three or more follicles >17 mm in diameter. Ovum pick-up followed 36 h after administration of 250 μg recombinant chorionic gonadotropin α (Ovitrelle®, Merck Serono, Darmstadt, Germany) by ultrasound-guided aspiration with a 17G needle (Cook, K-OSN-1730-B-90, Mönchengladbach, Germany) and an aspiration pressure of 120 mmHg.

The main reason for switching treatment protocols was the wish to change protocol, as pregnancy was not achieved.

### IVF Laboratory Management: Fertilization, Embryo Culture, and Embryo Transfer

Oocytes were inseminated by *in vitro* fertilization (IVF) in Sydney IVF Fertilization Medium (K-SIFM-20, Cook Medical) or by intracytoplasmic sperm injection (ICSI) in Sydney IVF Gamete Buffer (K-SIGB-50, Cook Medical). Oocytes were fertilized by ICSI in *n* = 67 cycles (*n* = 33 GnRH agonist and *n* = 34 GnRH antagonist cycles), TESE-ICSI in *n* = 6 cycles (*n* = 3 GnRH agonist and *n* = 3 GnRH antagonist cycles), IVF in *n* = 20 cycles (*n* = 11 GnRH agonist and *n* = 9 GnRH antagonist cycles), or IVF/ICSI splitting in *n* = 5 cycles (*n* = 2 GnRH agonist and *n* = 3 GnRH antagonist cycles).

In case of IVF oocytes were cultured for 16–20 h in a standard incubator until fertilization was checked on day 1. Pronuclear stage oocytes (PNs) were then placed individually in an EmbryoSlide (Vitrolife, Sweden) pre-filled and -equilibrated with Sydney IVF Cleavage Medium (K-SICM-20, Cook Medical) covered with paraffin oil (10100060A, Origio). In case of ICSI metaphase II (MII) oocytes were placed in an EmbryoScope immediately after the ICSI procedure.

Oocytes and embryos were cultured at 37°C in an atmosphere of 5.0% O_2_. In all incubators the CO_2_ concentration was set to adjust the pH of the culture medium within a range of pH= 7.25–7.35. Media change was performed on day 3 of culture by replacing 25 μl of the spent media with pre-equilibrated Sydney IVF Blastocyst Medium (K-SIBM-20, Cook Medical). After media change the EmbryoSlide was reinserted into the incubator and the culture was continued until transfer or vitrification.

Embryo transfers were scheduled for day 2 or 5 depending on the number of available PNs and the number of embryos intended for transfer. If day 2 or 5 transfers were not possible embryos were transferred on day 3 or 4. Due to national regulations (Embryo Protection Act) only a limited number of embryos may be cultured. Briefly, under the Embryo Protection Act a number of fertilized oocytes, which is expected to lead to 1–3 transferable blastocysts, can be cultured (33). Surplus PNs were cryopreserved.

Extended culture until day 4 or 5 was initiated if on the day of fertilization check more PNs were available than intended for transfer. In case of GnRH agonist treatments *n* = 38 cycles with an average of 4.0 embryos (min. 2, max. 6) were scheduled for extended culture, and *n* = 11 cycles with an average of 1.8 embryos (min. 1, max. 3) for day 2–3 culture. In case of GnRH antagonist treatments *n* = 36 cycles were scheduled for extended culture with an average of 3.9 embryos (min. 3, max. 5 embryos) and *n* = 12 cycles with an average of 1.8 embryos (min. 1, max. 2) for day 2–3 culture. One GnRH antagonist cycle did not produce an embryo.

Transfers were performed earlier than initially scheduled in some cases if the number of embryos suitable for transfer reached the number of embryos intended for transfer during the culture period (*n* = 3 GnRH agonist cycles, *n* = 6 GnRH antagonist cycles).

### Embryo Grading

Embryos were graded at recommended times as described previously (2, 34). Cleavage stage embryos were graded as A= stage-specific cell size and cytoplasmic fragmentation < 10%; B= stage-specific cell size and cytoplasmic fragmentation 10–25%; C= cell size not stage-specific and/or cytoplasmic fragmentation 26–50%; D= fragmentation > 50%. Blastocysts were scored according to Gardner et al. (35). For analysis embryos were grouped as good-quality embryos (GQE) or poor-quality embryos (PQE) as described previously (34). Good quality embryos were defined as 2–4-cell and grade A or B on day 2; 5–8-cell and grade A or B on day 3; 9–16-cell and grade A or B, compacting or fully compacted morula on day 4; blastocyst grade ≥3BB on day 5. Top quality embryos were defined as a subgroup of GQE with an early cleavage. Early cleavage was defined as the first mitotic division at 20–27 h after ICSI or 20–29 h after IVF as described by Balaban et al. (2).

### Time-Lapse Imaging

Embryos were time-lapse imaged up to the day of embryo transfer in 7 focal planes and a time interval set to 10 min. Morphokinetics were annotated as described by Ciray et al. (12) and are presented in hours (h) as mean (± standard deviation) and 95% confidence intervals or as median (quartiles 25–75) in boxplots. Cleavage times tn (*n* = 2–9), as well as the time of morula formation (tM) and blastulation (tSB, tB, and tEB) were normalized to the time of pronuclear fading (tPNf). In some cases, annotation of tPNf (*n* = 4 embryos in GnRH agonist cycles, *n* = 11 embryos in GnRH antagonist cycles) or other time-points was not possible. These cases were excluded from the analysis of morphokinetics. Embryonic cell cycles (ECC) were calculated as follows: ECC1= t2-tPB2, ECC2= t4-t2, ECC3= t8-t4. The synchronicity of cell divisions (s) was calculated as: s2= t4-t3 or s3= t8-t5.

### Statistics

All data were analyzed by pairwise exclusion using SPSS Versions 22, 23, and 25 (IBM, USA). Normal distribution was tested using Shapiro Wilks. Since most morphokinetic data were not normally distributed they were analyzed using Mann-Whitney *U* test. Categorial data were analyzed using Fisher's exact test. The significance level was set to *p* = 0.05. Boxplots were generated using SPSS.

### Ethics Approval and Consent to Participate

This study was approved by the University of Heidelberg Ethics Committee (S-649/2016).

## Results

### Study Population

The mean age (± standard deviation; minimum–maximum) of the patients in the GnRH agonist group was 35.7 ± 4.3 (25–42) years (*n* = 49) and 35.8 ± 4.0 (28–42) years (*n* = 49) in the GnRH antagonist group (*p* = 0.940) (Table 1). The mean Anti-Muellerian Hormone (AMH) level (± standard deviation) was 3.09 ± 2.69 (0.7–14.8) ng/ml (*n* = 43) (Table 1). The mean body mass index (BMI) was 22.98 ± 3.86 (17.58–33.30) kg/m^2^ (*n* = 48) (Table 1). There were *n* = 6 smokers among the female patients (*n* = 48) (Table 1).

**Table 1:** Baseline characteristics of the study population.

**Parameter**	**GnRH agonist**	**GnRH antagonist**	***n***	p^*^
Age	35.7 ± 4.3 years	35.8 ± 4.0 years	49	0.94
AMH	3.09 ± 2.69 (0.7–14.8) ng/ml		43	
BMI	22.98 ± 3.86 (17.58–33.30) kg/m^2^		48	
Smoking	6		48	
Protocol	GnRH agonist followed by GnRH antagonist: *n* = 31		49	
	GnRH antagonist followed by GnRH agonist: *n* = 18			
IVF	11	9	20	
ICSI	33	34	67	
Splitting IVF/ICSI	2	3	5	
TESE-ICSI	3	3	6	

* *Mann-Whitney U Test asymptotic significance (2-tailed)*.

The main diagnoses were pathological spermiogram [*n* = 20 (40.8%)], endometriosis [*n* = 10 (20.4%)], tubal/uterine factor sterility [*n* = 10 (20.4%)], idiopathic sterility [*n* = 6 (12.2%)] and PCOS [*n* = 3 (6.1%)].

The mean number of months that had passed between the two analyzed treatment cycles of a patient were 7.65 ± 8.57 (1–39) [mean ± standard deviation (min.–max.)]. The COS protocol was switched from GnRH agonist to antagonist in *n* = 31 patients and from GnRH antagonist to agonist in *n* = 18 patients (Table 1).

The average number of oocytes collected was *n* = 10.3 ± 4.5 in the GnRH agonist and *n* = 9.5 ± 4.1 in the GnRH antagonist group (*p* = 0.54) (Table 2). The maturation rate (the percentage of MII oocytes per total number of oocytes recovered) could be calculated for ICSI cycles and was 79.3% (299/377) in GnRH agonist and 73.6% (262/356) in GnRH antagonist cycles (*p* = 0.081) (Table 2). The overall fertilization rate was 55.6% (238/428) for GnRH agonist and 56.0% (209/373) for GnRH antagonist cycles (*p* = 0.943) (Table 2). The fertilization rate after ICSI treatment was 53.5% (160/299) in GnRH agonist and 55.0% (144/262) in GnRH antagonist treatments (*p* = 0.735) (Table 2). The fertilization rate after IVF treatments was 60.5% (78/129) in GnRH agonist and 58.6% (65/111) in GnRH antagonist cycles (*p* = 0.793) (Table 2).

**Table 2:** Number of oocytes recovered, fertilized and cultured *in vitro*.

**Variable**	**GnRH agonist**	**GnRH antagonist**	***p***
Oocytes [*n* (mean ± st.dev)]	506 (10.3 ± 4.5)	467 (9.5 ± 4.1)	0.540^**^
Maturation rate^†^	79.3% (299/377)	73.6% (262/356)	0.081^*^
Fertilization rate	55.6% (238/428)	56.0% (209/373)	0.943^*^
Fertilization rate (ICSI)	53.5% (160/299)	55.0% (144/262)	0.735^*^
Fertilization rate (IVF)	60.5% (78/129)	58.6% (65/111)	0.793^*^
IVF embryos cultured	23.3% (40/172)	27.6% (45/163)	
ICSI embryos cultured (incl. TESE-ICSI)	76.7% (132/172)	72.4% (118/163)	0.381^*^
TESE-ICSI embryos cultured	4.1% (7/172)	4.3% (7/163)	1.000^*^
Direct Cleavage	6.1% (10/163)	4.6% (7/152)	0.623^*^

*Direct Cleavage = t3-t2 ≤ 5 h*.

† *ICSI cycles only*.

* *Fisher's exact test (2-sided)*.

** *Mann-Whitney U Test asymptotic significance (2-tailed)*.

In GnRH agonist-cycles *n* = 172 embryos (*n* = 49 cycles) were cultured until day 2, *n* = 165 embryos (*n* = 44 cycles) until day 3, *n* = 143 embryos (*n* = 35 cycles) until day 4 and *n* = 80 embryos (*n* = 19 cycles) until day 5. In GnRH antagonist cycles *n* = 163 embryos (*n* = 48 cycles) were cultured until day 2, *n* = 146 embryos (*n* = 40 cycles) until day 3, *n* = 125 embryos (*n* = 31 cycles) until day 4 and *n* = 96 embryos (*n* = 24 cycles) until day 5. Extended culture until day 4 or 5 was aimed at in *n* = 38 cycles or *n* = 36 cycles (GnRH agonist or GnRH antagonist) and rescheduled to an earlier day due to poor embryo development in *n* = 3 cycles or *n* = 6 cycles (GnRH agonist or GnRH antagonist) (*p* = 0.302). The number of embryos cultured from IVF or ICSI treatments did not differ significantly between the study groups (Table 2). GnRH agonist cycles included *n* = 40 embryos (23.3%) from IVF and *n* = 132 embryos (76.7%) from ICSI treatments, whereas GnRH antagonist cycles included *n* = 45 embryos (27.6%) from IVF and *n* = 118 embryos (72.4%) from ICSI treatments (*p* = 0.381). Also, the number of embryos from TESE-ICSI treatments did not differ between the study groups (Table 2). GnRH agonist cycles included *n* = 7 embryos (4.1%) from TESE-ICSI treatments, whereas GnRH antagonist cycles included *n* = 7 embryos (4.3%) from TESE-ICSI treatments (*p* = 1.000).

### Quality of Embryo Morphology in GnRH Agonist vs. Antagonist Cycles

There was no significant difference in the number of good quality embryos (GQE) and poor quality embryos (PQE) in GnRH agonist vs. antagonist cycles (Table 3). On day 2 GnRH agonist cycles included *n* = 116 (67.4%) GQE and *n* = 56 (32.6%) PQE, whereas GnRH antagonist cycles included *n* = 118 (72.4%) GQE and *n* = 45 (27.6%) PQE (*p* = 0.342). On day 3 GnRH agonist cycles included *n* = 92 (55.8%) GQE and *n* = 73 (44.2%) PQE, whereas GnRH antagonist cycles included *n* = 84 (57.5%) GQE and *n* = 62 (42.5%) PQE (*p* = 0.819). On day 4 GnRH agonist cycles included *n* = 78 (54.5%) GQE and *n* = 65 (45.5%) PQE, whereas GnRH antagonist cycles included *n* = 72 (57.6%) GQE and *n* = 53 (42.4%) PQE (0.624). On day 5 GnRH agonist cycles included *n* = 22 (27.5%) GQE and *n* = 58 (72.5%) PQE, whereas GnRH antagonist cycles included *n* = 31 (32.3%) GQE and *n* = 65 (67.7%) PQE (*p* = 0.514). On the day of embryo transfer GnRH agonist cycles included *n* = 61 (35.5%) GQE and *n* = 111 (64.5%) PQE, whereas GnRH antagonist cycles included *n* = 61 (37.4%) GQE and *n* = 102 (62.6%) PQE (*p* = 0.734).

**Table 3:** Numbers of embryos with good quality (GQE) or top quality (TQE) from patients treated with GnRH agonists depending on the day of morphology assessment.

**Day**	**Protocol**	**GQE [*n*/*n* (total) (%)]**	p^*^	**TQE (*n*/*n* (total) (%)]**	p^*^
2	GnRH agonist	116/172 (67.4%)	0.342	60/170 (35.3%)	0.820
	GnRH antagonist	118/163 (72.4%)		60/163 (36.8%)	
3	GnRH agonist	92/165 (55.8%)	0.819	51/165 (30.9%)	0.716
	GnRH antagonist	84/146 (57.5%)		48/146 (32.9%)	
4	GnRH agonist	78/143 (54.5%)	0.624	46/143 (32.2%)	1.000
	GnRH antagonist	72/125 (57.6%)		41/125 (32.8%)	
5	GnRH agonist	22/80 (27.5%)	0.514	17/80 (21.3%)	0.720
	GnRH antagonist	31/96 (32.3%)		23/96 (24.0%)	
Final day	GnRH agonist	61/172 (35.5%)	0.734	28/170 (16.5%)	1.000
	GnRH antagonist	61/163 (37.4%)		27/163 (16.6%)	

* *Fisher's exact test (2-sided)*.

Also, the number of top quality embryos (TQE), here defined as a subgroup of GQE, did not differ significantly between GnRH agonist vs. antagonist cycles (Table 3). On day 2 GnRH agonist cycles included *n* = 60 TQE (35.3%), whereas GnRH antagonist cycles included *n* = 60 TQE (36.8%) (*p* = 0.820). On day 3 GnRH agonist cycles included *n* = 51 (30.9%) TQE, whereas GnRH antagonist cycles included *n* = 48 (32.9%) TQE (*p* = 0.716). On day 4 GnRH agonist cycles included *n* = 46 (32.2%) TQE, whereas GnRH antagonist cycles included *n* = 41 (32.8%) TQE (*p* = 1.000). On day 5 GnRH agonist cycles included *n* = 17 (21.3%) TQE, whereas GnRH antagonist cycles included *n* = 23 (24.0%) TQE (*p* = 0.720). On the day of embryo transfer GnRH agonist cycles included *n* = 28 (16.5%) TQE, whereas GnRH antagonist cycles included *n* = 27 (16.6%) TQE (*p* = 1.000).

### Embryo Morphokinetics of GnRH Agonist vs. Antagonist Cycles

**Figure 1:**
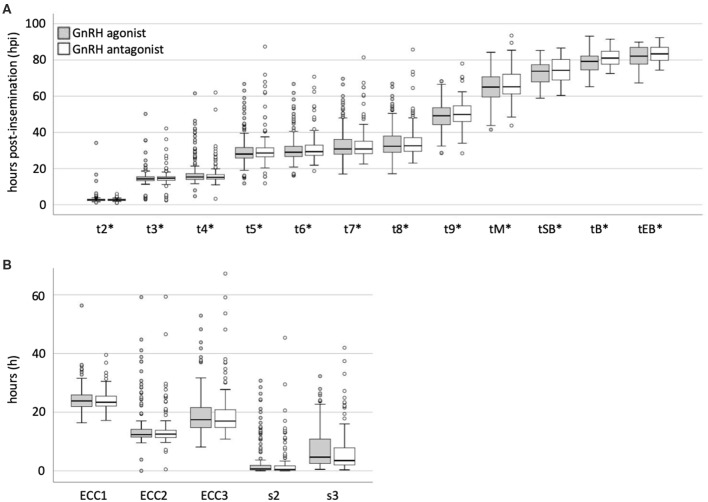
Comparison of morphokinetic timings of embryos from patients treated with GnRH agonists or antagonists. **(A)** Morphokinetic timings (*hours post-insemination normalized to the time of pronuclear fading) and **(B)** times (hours) of developmental periods of embryos from patients treated with GnRH agonists or antagonists. Boxplots were generated using SPSS.

There was no significant difference in the rate of direct cleavage (DC, t3-t2 ≤ 5 h) between the study groups [DC (GnRH agonist) = 10/163 (6.1%) and DC (GnRH antagonist) = 7/152 (4.6%) (*p* = 0.623)] (Table 2). We found no statistically significant difference between the timings of the analyzed morphokinetic parameters between the study groups (Table 4, Figures 1A,B and Supplementary Figure 1). The duration of the first embryonic cell cycle (ECC1= t2-tPB2) was 24.6 ± 4.7 h in GnRH agonist cycles (*n* = 129) and 24.2 ± 3.7 h in GnRH antagonist cycles (*n* = 91, *p* = 0.66, Table 4). The duration of the second embryonic cell cycle (ECC2= t4-t2) was 14.8 ± 7.5 h in GnRH agonist cycles (*n* = 158) and 13.9 ± 6.2 h in GnRH antagonist cycles (*p* = 0.65, *n* = 145, Table 4). The duration of the third embryonic cell cycle (ECC3 = t8-t4) was 19.7 ± 7.8 h in GnRH agonist cycles (*n* = 121) and 19.9 ± 9.2 h in GnRH antagonist cycles (*p* = 0.72, *n* = 113, Table 4). Also, there was no significant difference in the synchrony of cell divisions during ECC2 (s2 = t4-t3, *p* = 0.24) or ECC3 (s3 = t8-t5, *p* = 0.09, Table 4).

**Table 4:** Morphokinetic timings (hours) of embryos from patients treated with GnRH agonists or GnRH antagonists.

	**GnRH agonist**			**GnRH antagonist**			
	**Mean±Std.Dev. (hours)**	**CI 95% (hours)**	***n***	**Mean ± Std.Dev. (hours)**	**CI 95% (hours)**	***n***	p^*^
t2-tPNf	3.1 ± 2.9	2.7–3.6	167	2.7 ± 0.6	2.6–2.8	152	0.30
t3-tPNf	14.7 ± 4.9	13.9–15.5	161	14.9 ± 4.4	14.1–15.6	145	0.38
t4-tPNf	17.8 ± 7.8	16.5–19.0	156	16.7 ± 6.4	15.7–17.8	138	0.70
t5-tPNf	29.8 ± 8.7	28.4–31.2	147	30.9 ± 10.6	29.1–32.8	126	0.38
t6-tPNf	30.9 ± 8.4	29.5–32.3	137	31.6 ± 8.3	30.0–33.1	118	0.37
t7-tPNf	33.7 ± 9.3	32.1–35.3	132	33.8 ± 9.9	32.0–35.6	115	0.91
t8-tPNf	35.1 ± 9.3	33.4–36.8	119	35.4 ± 10.3	33.4–37.4	106	0.91
t9+ -tPNf	48.9 ± 8.0	47.3–50.4	102	50.3 ± 7.9	48.7–51.9	92	0.35
tM-tPNf	64.5 ± 8.4	62.7–66.3	88	66.7 ± 9.5	64.6–68.9	80	0.39
tSB-tPNf	73.0 ± 6.4	71.4–74.6	62	74.8 ± 6.8	73.0–76.6	59	0.18
tB-tPNf	78.9 ± 6.7	77.0–80.8	49	81.2 ± 5.0	79.7–82.7	45	0.08
tEB-tPNf	81.3 ± 6.0	79.2–83.3	36	83.2 ± 4.5	81.6–84.9	32	0.24
ECC1 (=t2-tPB2)	24.6 ± 4.7	23.8–25.4	129	24.2 ± 3.7	23.4–25.0	91	0.66
ECC2 (=t4-t2)	14.8 ± 7.5	13.6–16.0	158	13.9 ± 6.2	12.9–15.0	145	0.65
ECC3 (=t8-t4)	19.7 ± 7.8	18.3–21.1	121	19.9 ± 9.2	18.2–21.6	113	0.72
s2 (=t4-t3)	3.2 ± 6.1	2.2–4.1	158	2.5 ± 5.7	1.6–3.4	145	0.24
s3 (=t8-t5)	7.6 ± 7.3	6.3–8.9	121	6.7 ± 7.9	5.2–8.2	113	0.09

*Presented are the mean, standard deviation (std. dev.) and the 95% confidence interval (CI)*.

* *Mann-Whitney U Asymp. Sig. (2-tailed)*.

## Discussion

Although COS is described as a possible confounder for embryo morphokinetics, its influence on embryo development is still unclear (12). Several studies investigated differences in the outcomes between GnRH agonist protocols with hCG-trigger vs. GnRH antagonist protocols with GnRH agonist-trigger.

In this study we retrospectively analyzed if there are intraindividual differences in embryo quality and morphokinetics after switching the type of GnRH analog used, i.e., GnRH agonist or antagonist, in consecutive treatment cycles of the same patients. In this study we only used recombinant hCG to trigger ovulation in both GnRH agonist and GnRH antagonist cycles.

When comparing oocyte yield, significantly less cumulus-oocyte complexes (COCs) were reported in GnRH antagonist cycles, but no difference in the number of metaphase II (MII) oocytes (30, 36). In our study a switch in the GnRH analog did not significantly change the number of COCs retrieved. Also, we did not observe any significant difference in the number of MII oocytes in ICSI cycles.

Bosch et al. described, that COS may impact on the developmental competence of embryos (25). In our study, the proportion of GQEs, as well as TQEs, here defined as a subgroup of GQE, decreased throughout the culture period. However, we did not find a significant difference in the proportion of GQEs or TQEs between the study groups.

Concerning developmental rates Muñoz et al. described faster cleavage t2-t5 in cycles with GnRH antagonists and GnRH agonist-trigger compared to GnRH agonist cycles with hCG-trigger, but the embryo quality in terms of morphokinetics was not different (31). Mumusoglu on the other hand did not find differences in embryo morphokinetics in GnRH agonist vs. antagonist protocols in an analysis of PGT cycles (37). In agreement with the study of Mumusoglu et al. we found no difference in the morphokinetics of embryos from GnRH agonist vs. antagonist cycles (37).

Focusing on GnRH antagonist cycles, Gurbuz et al. found that early pre-implantation development was faster in cycles with GnRH agonist-trigger compared to cycles with hCG-trigger, specifically tPB2, tPNf, t2, t3, t5, and t6 (38). This finding revealed that the method used to trigger final oocyte maturation might affect oocyte competence and early pre-implantation embryo development. In our study, ovulation was triggered with hCG in all cycles. Therefore, the mode of ovulation trigger does not represent a confounder for the two study groups in our analysis.

The studies discussed above used interindividual comparison, which is possibly prone to confounders. In the present study we aimed to investigate if intraindividual embryo morphokinetics are affected by switching the type of GnRH analog used (GnRH agonist vs. GnRH antagonist) in consecutive treatment cycles of COS, in order to eliminate possible confounders that may impact in an interindividual study design. Using a similar study design Lai et al. also did not find a statistically significant difference in the number of oocytes recovered, the fertilization rate and embryo quality between the GnRH agonist long protocol and the GnRH antagonist protocol with hCG-trigger (39).

Supporting our findings on early embryo quality and morphokinetics, a recent review found no difference between the GnRH antagonist and the long GnRH agonist protocol in COS regarding live birth and miscarriage rates (32).

Therefore, our study supports the flexible use of GnRH agonist or antagonist protocols in a clinical setting. Many IVF units change the stimulation protocol if pregnancy was not achieved. As our study did not show any significant difference in embryo morphokinetics between the study groups, the decision to switch the stimulation protocol can be based on patient's individual needs.

This provides an advantage considering the highly individual patient characteristic that may guide treatment decisions, particularly in efforts to reduce the incidence of ovarian hyperstimulation syndrome (OHSS) in women with high ovarian reserve (32).

The main limitations of our study are its retrospective design, as well as the small sample size, which implicates the need for further confirmation studies. This study focused on embryo morphology and morphokinetics. Thus, these data do not necessarily reflect the life birth rate. Furthermore, as only hCG was used as a trigger, the results may be different when another trigger is applied. A definite strength is the intraindividual approach, excluding individual confounders.

## Conclusions

In conclusion, we did not find significant differences in the quality of embryo morphology and embryo morphokinetics after switching the type of GnRH analog used for COS in consecutive treatment cycles of the same patient. This finding supports the flexible use of GnRH analogs to optimize patient treatment for COS.

## Data Availability Statement

The datasets generated for this study are available on request to the corresponding author.

## Ethics Statement

The studies involving human participants were reviewed and approved by University of Heidelberg Ethics Committee (S-649/2016). Written informed consent for participation was not required for this study in accordance with the national legislation and the institutional requirements.

## Author Contributions

AF and JD were involved in designing the study, acquisition, analysis and interpretation of data, as well as drafting and revising the manuscript. AG was involved in designing the study, analysis and interpretation of data, drafting and revising the manuscript. FB was involved in designing the study, acquisition of data, and revising the manuscript. JL was involved in the acquisition of data. KH and VH were involved in drafting and revising the manuscript. TS was involved in designing the study, interpreting the results, and revising the manuscript. All authors read and approved the final manuscript.

## Conflict of Interest

The authors declare that the research was conducted in the absence of any commercial or financial relationships that could be construed as a potential conflict of interest.
