# Supplementary figures and images for: Intraindividual Embryo Morphokinetics Are Not Affected by a Switch of the Ovarian Stimulation Protocol Between GnRH Agonist vs. Antagonist Regimens in Consecutive Cycles
Source: Front Endocrinol (Lausanne). 2020 Apr 28;11:246. doi: 10.3389/fendo.2020.00246 (PMC7198727; doi:10.3389/fendo.2020.00246)

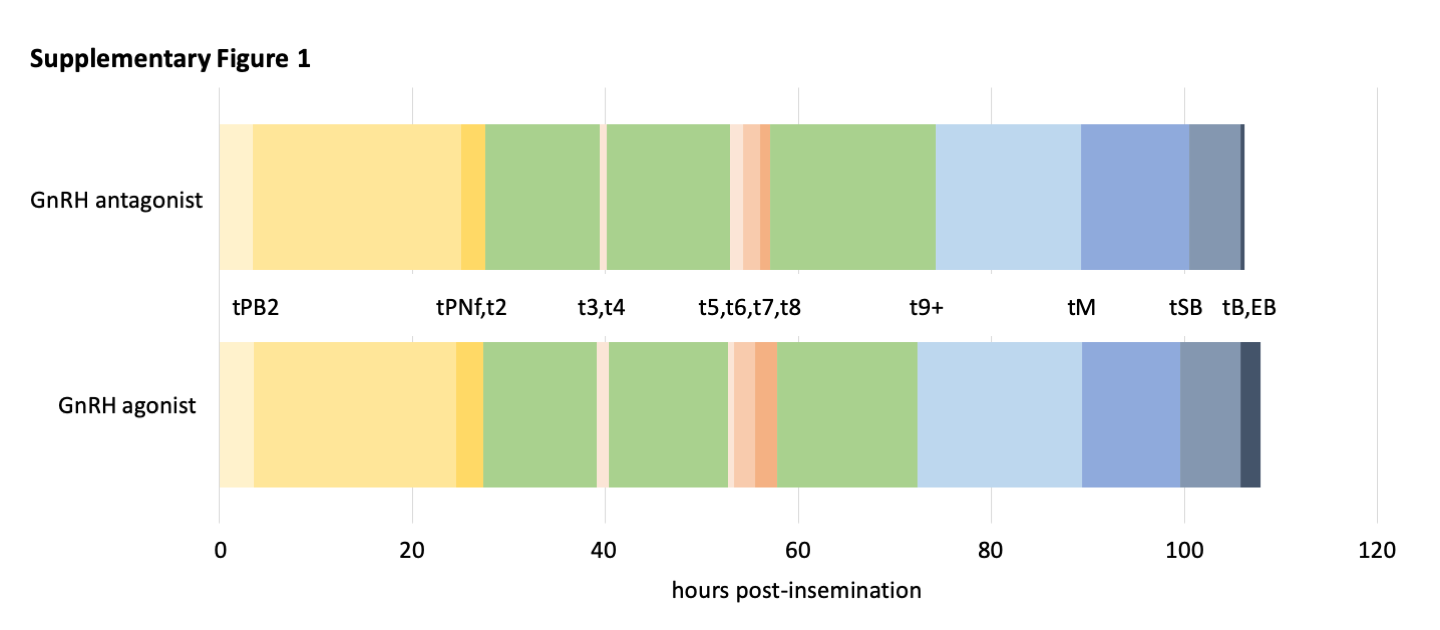

Supplement: Supplementary file 1 [file Image_1.tiff]
